# SQUID magnetoneurography: an old-fashioned yet new tool for noninvasive functional imaging of spinal cords and peripheral nerves

**DOI:** 10.3389/fmedt.2024.1351905

**Published:** 2024-04-16

**Authors:** Yoshiaki Adachi, Shigenori Kawabata

**Affiliations:** ^1^ Applied Electronics Laboratory, Kanazawa Institute of Technology, Kanazawa, Japan; ^2^ Department of Advanced Technology in Medicine, Tokyo Medical and Dental University, Tokyo, Japan; ^3^ Section of Orthopaedic and Spine Surgery, Graduate School of Tokyo Medical and Dental University, Tokyo, Japan

**Keywords:** magnetoneurography, magnetospinography, biomagnetism, superconducting quantum interference device (SQUID), neuropathy, spinal cord, peripheral nerves

## Abstract

We are engaged in the development and clinical application of a neural magnetic field measurement system that utilizes biomagnetic measurements to observe the activity of the spinal cord and peripheral nerves. Unlike conventional surface potential measurements, biomagnetic measurements are not affected by the conductivity distribution within the body, making them less influenced by the anatomical structure of body tissues. Consequently, functional testing using biomagnetic measurements can achieve higher spatial resolution compared to surface potential measurements. The neural magnetic field measurement, referred to as magnetoneurography, takes advantage of these benefits to enable functional testing of the spinal cord and peripheral nerves, while maintaining high spatial resolution and noninvasiveness. Our magnetoneurograph system is based on superconducting quantum interference devices (SQUIDs) similar to the conventional biomagnetic measurement systems. Various design considerations have been incorporated into the SQUID sensor array structure and signal processing software to make it suitable for detecting neural signal propagation along spinal cord and peripheral nerve. The technical validation of this system began in 1999 with a 3-channel SQUID system. Over the course of more than 20 years, we have continued technological development through medical-engineering collaboration, and in the latest prototype released in 2020, neural function imaging of the spinal cord and peripheral nerves, which could also be applied for the diagnosis of neurological disorders, has become possible. This paper provides an overview of the technical aspects of the magnetoneurograph system, covering the measurement hardware and software perspectives for providing diagnostic information, and its applications. Additionally, we discuss the integration with a helium recondensing system, which is a key factor in reducing running costs and achieving practicality in hospitals.

## 1 Introduction

Biomagnetic field measurement is a promising tool for noninvasive investigation of human neural activity (1, 2). Weak magnetic fields evoked by electric current induced by activity of neurons or muscles penetrate through body tissues such as lipids, bones, and skins, and can be detected by multiple highly sensitive magnetic flux sensors arranged along the body surface. The original neural activity is noninvasively determined by analysis of the obtained magnetic field data. As applications of biomagnetic field measurements, magnetoencephalography (MEG) (3) and magnetocardiography (MCG) (4, 5), which measure the biomagnetic fields of the brain and heart to provide functional information, respectively, have already been commercialized as medical devices and introduced to research institutions and hospitals.

In the fields of orthopaedic surgery and neurology, there is a strong demand for methods to noninvasively test the signal propagation functions of nerves. For example, when the spinal cord is locally compressed due to conditions such as degenerative spinal diseases, nerve signal transmission is interrupted, leading to neurological symptoms like limb numbness and fine motor skill impairments. While these conditions can be treated by surgically relieving the pressure on the spinal cord, it is crucial to identify the affected area preoperatively to minimize patient burden during surgery and to ensure effective outcomes from the procedure. The identification of the affected area in spinal cord diseases has conventionally relied on a combination of clinical symptoms, physical examinations, and neurological assessments, supplemented by imaging findings from magnetic resonance images (MRIs) or x-ray computed tomographies (CTs). However, medical doctors have often expressed frustration with false-positive results, as these imaging abnormalities are not always correlated with clinical symptoms (6). To reduce the risk of false-positive results, functional information through neurophysiological spinal function diagnosis is significant. Traditionally, neural function tests have widely employed potential measurement tests, which attach electrodes to the body surface and detect variations in potential distribution associated with neural electrical activity. However, the potentials that appear on the body surface are severely influenced by anatomical structures of tissues with deferent conductivities, such as bones and lipids. As a result, it has been challenging to obtain functional information from nerves enclosed by bones, like the spinal cord, with the spatial resolution required for diagnosis. Consequently, invasive measures, such as the insertion of an epidural catheter electrodes into the spinal canal, had to be taken to obtain electrophysiological signals near the spinal cord (7).

On the other hand, the magnetic permeability within the body remains largely constant, nearly equivalent to the permeability of atmosphere, regardless of variations in body tissues. As a result, the magnetic field distribution observed at the body surface is less susceptible to the influence of anatomical structures of the tissues unlike electric potentials. Therefore, functional assessments using biomagnetic measurements can achieve higher spatial resolution compared to surface potential measurements. Magnetoneurographs, which include magnetospinographs, magnetically capture the propagation of the neural signals along axons. Historically, the first magnetoneurography was reported in 1980 (8) with a superconducting quantum interference device (SQUID) to record neural activity of a frog sciatic nerve. Alongside advancements in SQUID technology, various studies have reported on biomagnetic measurements taken from the cervical spinal cord (9–11). However, there has been limited focus on the practical clinical applications of spinal cord magnetic field measurements. The amplitude of observed magnetospinographic signals at the body surface is significantly small, owing to the source of these signals being located at a relatively deep position within the body. Consequently, traditional SQUID biomagnetometers face challenges in acquiring reproducible signals with adequate signal-to-noise ratios.

**Figure 1:**
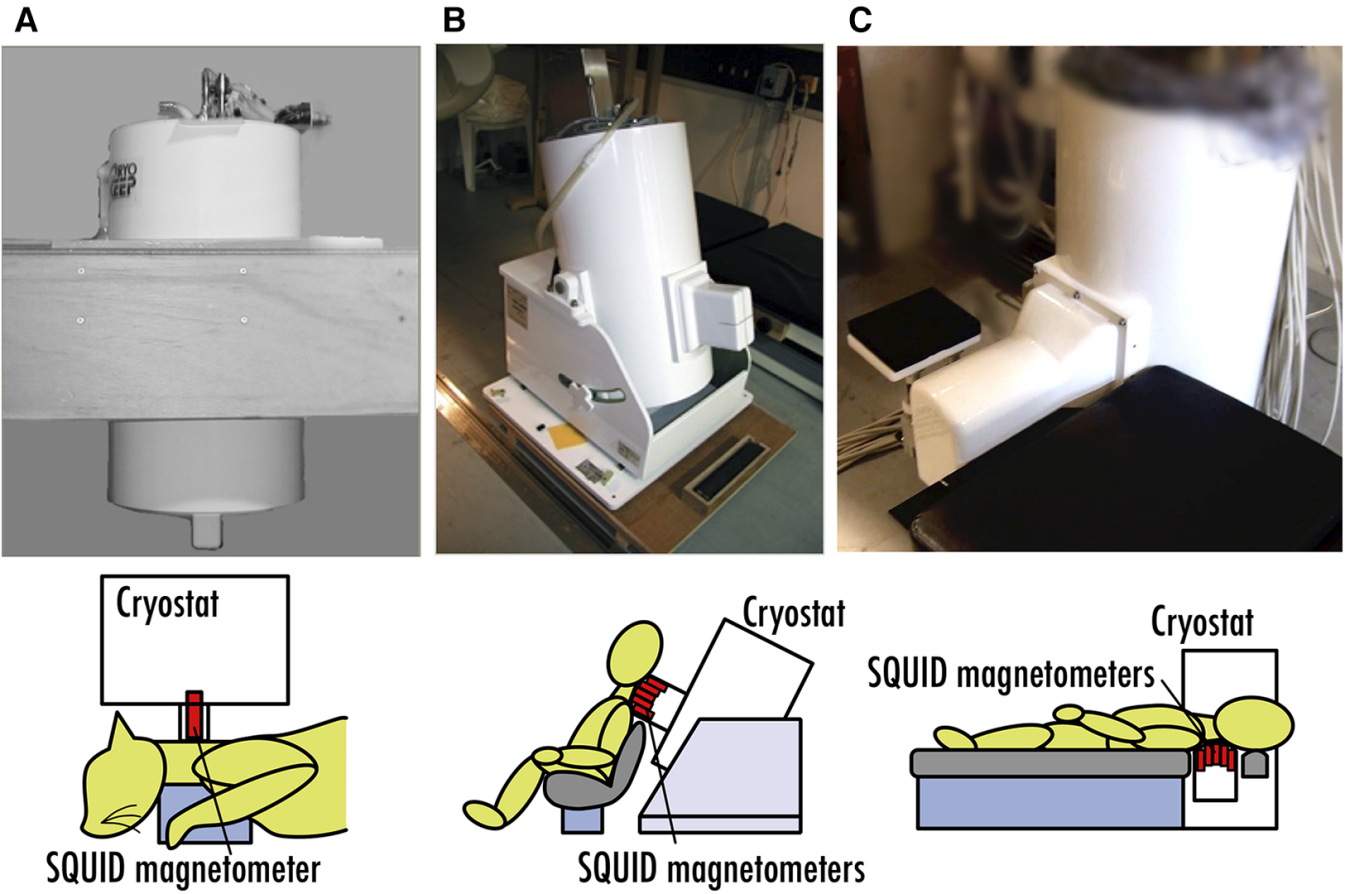
Chronicle of SQUID systems for magnetoneurography research. (**A**) 3-ch vector-type SQUID system for animal experiment in 1999. (**B**) 30-ch SQUID measurement system for seated subjects developed in 2004. (**C**) SQUID measurement system for supine subjects developed in 2007.

We are working on the development and clinical applications of a neuromagnetic measurement system by applying biomagnetic measurements, starting from building a SQUID system optimized for magnetoneurography from scratch. Figure 1 depicts the chronicle of the SQUID systems for our research of magnetoneurography. Our research and development project for magnetoneurography initiated in 1999 with technology verification through animal experiments detecting cervical spinal cord evoked field signals from cats using a 3-ch vector-type SQUID system (12), and success was achieved in 2004 in detecting spinal cord evoked magnetic field signals traveling along the cervical spinal cord of humans for the first time using a 30-ch SQUID measurement system adapted for seated subjects (13). In 2007, a measurement system known as the “supine model” was developed to detect spinal cord evoked magnetic fields from the dorsal side of supine subjects, targeting clinical applications in hospitals (14). Continuous efforts have been made since then to improve the device and enhance its clinical applications. Currently, not only spinal cord but also peripheral nerve functional imaging has become possible (15). Collaborating with a private company, we are actively working towards the practical application of this technology as a medical device.

## 2 Magnetoneurography by SQUID magnetometers

**Figure 2:**
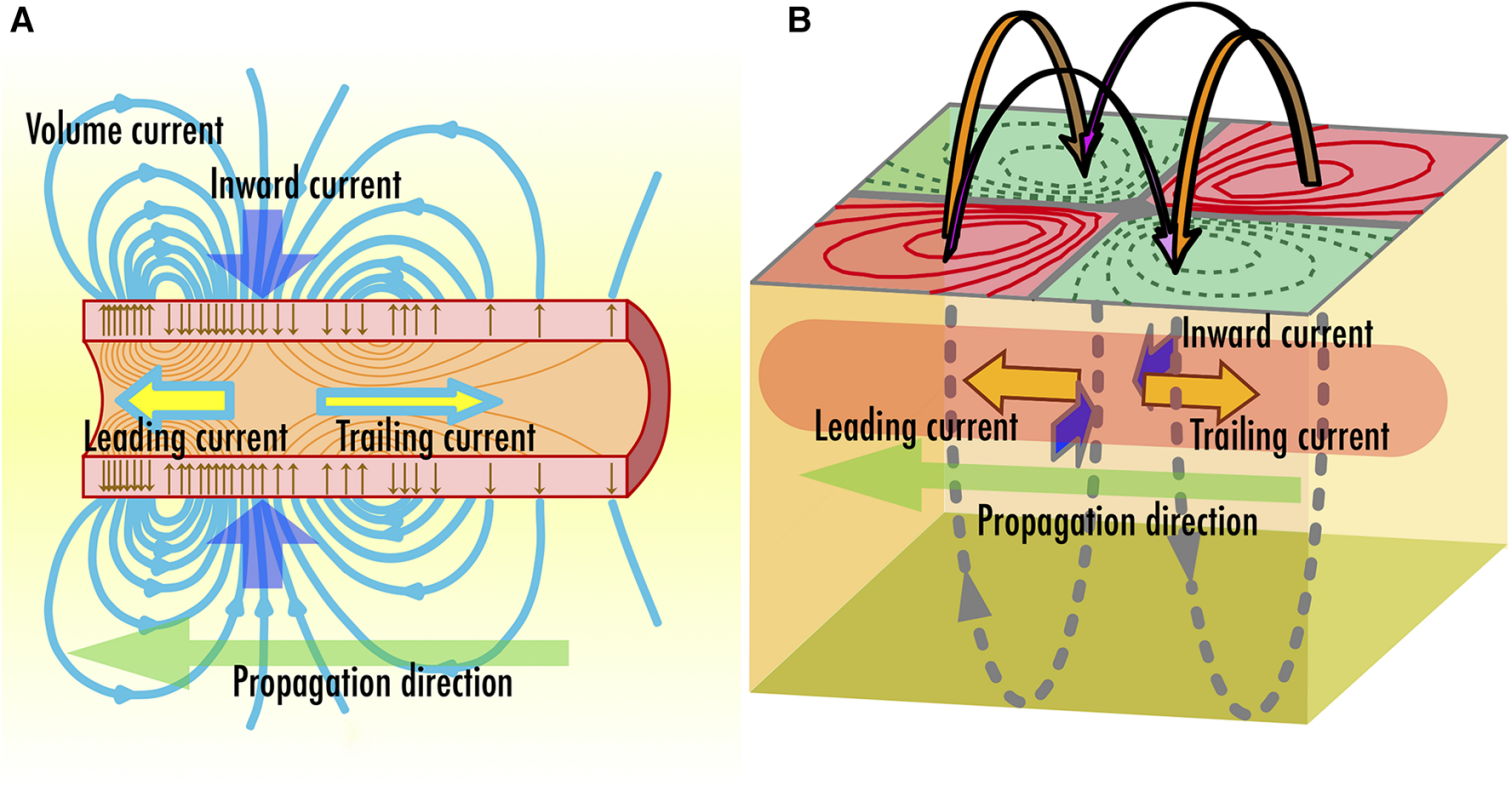
(**A**) Schematic illustration of two intra-axonal current components, which are leading current and trailing current, and inward volume current accompanied with neural signal propagation along axon. (**B**) Direction of magnetic fields observed along the body surface.

In conventional MEG, the observed brain magnetic fields are commonly attributed to postsynaptic potentials (3). Conversely, our neuromagnetic measurement systems target magnetic fields traveling along the spinal cord and peripheral nerves, which originate from action potentials propagating along axons. Figure 2A illustrates the schematic distribution of intracellular and extracellular currents at an active site on the axon where an action potential is generated (16). Within the nerve cell, bidirectional current pairs are established along the axon, resulting from the opening and closing of ion channels as well as osmotic ion flows through the cellular membrane. The intracellular current component that precedes in the same direction as the propagation of a neural signal is referred to as “leading current,” while the component that follows in the opposite direction is called “trailing current.” In the extracellular space, ion flows are generated over an extensive area to compensate the intracellular currents, commonly referred to as “volume currents.” Especially, the volume current flowing into the axon, which is positioned between the leading current and trailing current, is referred to as the “inward current.” Both these intracellular and volume currents act as the source of the spinal cord and peripheral nerve magnetic fields. Upon measuring the magnetic field distribution caused by these currents at the body surface, a quadrupole-like pattern emerges, consisting of two pairs of magnetic sources and sinks, as schematically depicted in Figure 2B. Notably, the frequency response of action potentials is broader compared to postsynaptic potentials; while brain magnetic field signals typically fall below a few hundred hertz, the frequency range for spinal and peripheral nerve magnetic fields extends from 100 Hz to several kHz (17, 18).

The magnetic fields emanating from the spinal cord and peripheral nerve observable at the body surface are quite diminutive in intensity even when compared to brain or cardiac signals. They are only about one-billionth to one-tenth-billionth intensity of the Earth's magnetic field. Consequently, their detection typically necessitates the use of highly sensitive magnetic flux sensors employing SQUIDs, hereafter referred to as SQUID magnetometers. Recent advances have brought attention to alternative magnetic sensors such as optically pumped atomic magnetometers, flux gates, and magnetoresistive-device-based sensors operating at room temperature, as they offer relatively low-cost solutions for biomagnetic measurements (19–21). While these sensors hold promise for substantially expanding the applicability of biomagnetic measurements, they currently fall short of SQUID magnetometers in terms of magnetic field resolution, bandwidth, and usability. As long as cooling can be achieved, SQUID magnetometers remain the most suitable choice for measuring magnetic fields from spinal cord and peripheral nerves. This section briefly outlines the application of SQUIDs in neuromagnetic measurement systems.

**Figure 3:**
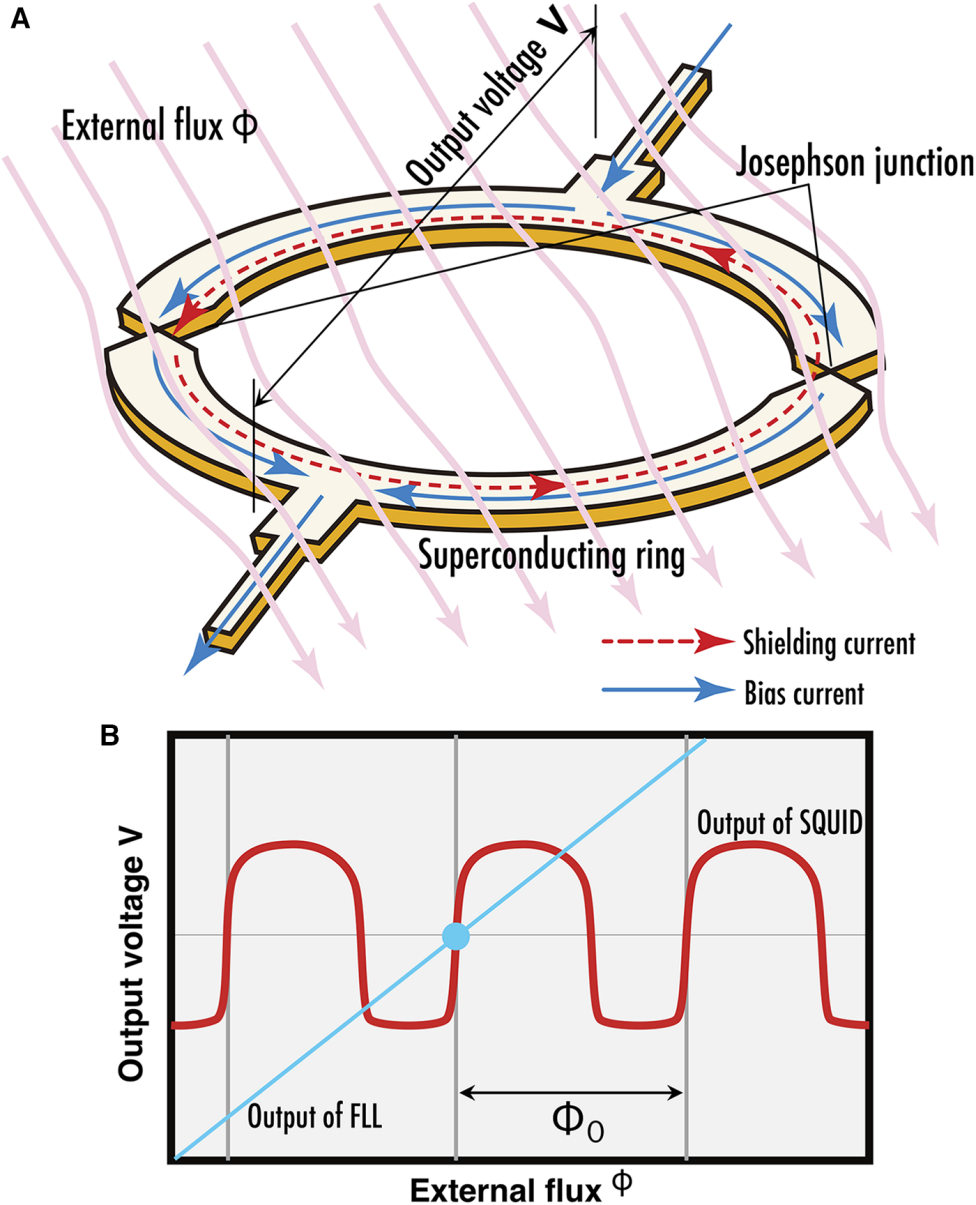
(**A**) Current flow along SQUID ring when exposed to external magnetic flux. (**B**) Periodic flux-voltage characteristics of SQUID output (red curve) and output linearized by FLL circuit (cyan line).

The SQUID magnetometers applied in neuromagnetic measurement systems are fundamentally based on low-temperature superconducting DC SQUIDs as well as those used in MEG and MCG (22). A DC SQUID comprises a ring-shaped superconducting structure featuring two weak links known as Josephson junctions, as illustrated in Figure 3A. When an external magnetic flux attempts to penetrate the ring, a shielding current flows to negate the external flux. By applying a bias current exceeding the superconducting critical current to the ring, a voltage corresponding to the shielding current emerges across the junctions, enabling its function as a magnetic flux sensor. Given the small size of individual SQUID rings, it is common measure in biomagnetic measurements to couple with detection coils made of superconducting wire to enhance sensitivity of the external flux. Differential pick up coils, considering of two oppositely wound coils placed at a fixed distance and connected in series, are frequently utilized. Environmental magnetic fields, which are regarded as noise in biomagnetic measurements, are considered uniform due to their significant distance from the magnetic source and are thus canceled out when passing through the oppositely wound coils. Conversely, magnetic fields originating from nearby sources produce different flux penetrations through the closer and farther coils, and this difference is detected by the SQUID.

The voltage response of a SQUID relative to external magnetic flux exhibits a periodic, nonlinear curve characterized by periodicity corresponding to a flux quantum as depicted by a red curve in Figure 3B. The output of the SQUID is interfaced with a flux locked loop (FLL), a specialized circuit that employs a series of amplifiers, integrators, and voltage-to-current converters, alongside a coil to feed the output back as magnetic flux to the SQUID (23). This feedback mechanism is balanced to nullify any variations in the magnetic flux detected by the SQUID, effectively linearizing its output. The feedback quantity is regarded as the circuit's output signals. This is analogous to placing an object on one pan of a balance scale and adding weights to the opposite pan until the needle points to zero; the mass of the object is then determined by the mass of the weights. Such method of measuring physical quantities is called null method. By adopting this methodology, the inherently nonlinear and periodic output characteristics of the SQUID are linearized, thereby enhancing its dynamic range for magnetic flux detection as depicted by a cyan line in Figure 3B. In recent years, there has been an advancement towards the digitization of FLLs, from integrators to voltage-to-current converters.

## 3 Signal processing and magnetic source analysis for magnetoneurography

### 3.1 Magnetic source analysis based on spatial filter technique

In conventional MEG, it is often assumed that bundles of neurons with the same orientation in a specific brain region are activated synchronously. The current appearing in the active site is represented by a mathematical model known as an equivalent current dipole for magnetic source analysis (24). A current dipole is defined as a current flowing in an infinitesimally small volume at a point and is characterized by parameters such as position, direction, and intensity (moment). The unit of the current dipole moment is the ampere-meter (Am), which is the product of current and length. Numerical optimization techniques are employed to estimate the parameters of the current dipole that minimize the difference between the theoretical magnetic field distribution generated by the dipole and the measured magnetic field distribution. This magnetic source analysis method is referred to as the equivalent current dipole method.

**Figure 4:**
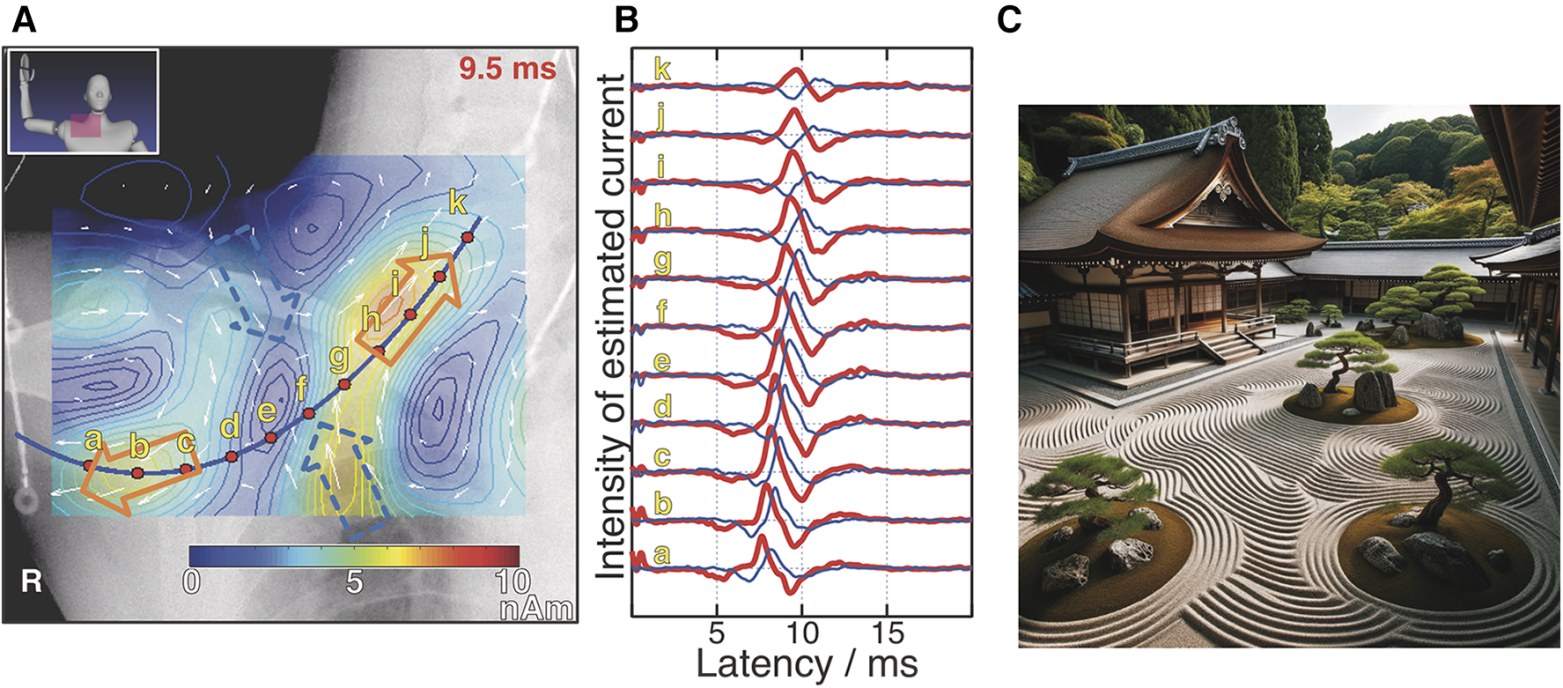
Example of magnetic source analysis in magnetoneurography (**A**) pseudo-color map indicates current distribution reconstructed based on magnetic field distribution captured by magnetoneurograph. Magnetic field was induced by right median nerve stimulation at wrist and captured in adjacent to right clavicle. Blue curve was neural pathway configured based on x-ray image. Red dots indicate position of virtual electrodes. Orange and blue hollow arrows indicate orientation of intra-axon current and inward volume current, respectively. (**B**) Estimated waveforms at each virtual electrode. Red and blue curves represent waveforms of parallel and inward perpendicular current components to neural pathway, respectively. Labels a–k are corresponding to those in (**A**). (**C**) Japanese traditional rock garden (sekitei). (**A,B**) are excerpts from reference (29). (**C**) was drawn by DALL-E-3.

When applying the equivalent current dipole method, commonly used in MEG, to the analysis of magnetic fields from the spinal cord proved challenging for obtaining sufficient information for lesion diagnosis (25). As discussed in the previous section, the sources of these magnetic fields originate from opposing intracellular currents formed along active sites on the axons and compensatory volume currents. While the equivalent current dipole method models and analyzes these intracellular currents, animal studies have shown that volume currents significantly affected by lesions also play a crucial role in diagnosis. Therefore, a magnetic source analysis based on spatial filtering method is employed to estimate a broader current distribution that includes volume currents (26). In this approach, the region of interest where magnetic sources exist is divided into *M* grids, and current elements are placed in each grid. Assuming that this group of current elements ***s* **= (*s*_0_,…, *s_M_*_–1_) can model the current distribution caused by neural electrical activity, we aim to estimate ***s*** or the direction and magnitude of each current element, from the obtained magnetic field distribution. When a particular ***s*** is assumed, the theoretical magnetic field signal vector ***b* **= (*b*_0_,…, *b_N_*_–1_) obtained from an array of *N* magnetic sensors can be expressed as ***b* **= **Ls**, where **L** is the lead field matrix that represents the sensitivity of each sensor to the corresponding current elements. In actual measurements, noise ***n*** should be considered, making it ***b***’ = **Ls **+ ***n***. The group of current elements is estimated using the measured ***b***’ and a weight matrix **W** derived from **L**, as ***s***’ = **W*b***’. In the measurement examples shown in Section 5, the unit gain constraint recursive null steering (UGRENS) beamformer is applied for the calculation of **W** (27, 28). Each current element has the same dimensional unit as a current dipole but is not necessarily modeling any specific neural activity. In other words, while individual current elements may not have a specific meaning, collectively they represent the current distribution across the region of interest. Overlaying the estimated current distribution onto anatomical images obtained from x-ray or MRI allows for the interpretation of what neural activities are presented by each current element as shown in Figure 4A (29). This is analogous to the concept in Japanese traditional rock garden (sekitei), where although each individual stone may not possess inherent meaning on its own, the overall distribution of stones represents significance and function (Figure 4C). Furthermore, software has been developed to display the temporal changes in estimated current waveforms enable the calculation of neural conduction velocities and reveal irregularities in the waveform itself, offering valuable diagnostic information regarding the site of lesion. Notably, the function of placing virtual current sensors (akin to virtual electrodes) along a neural pathway configured based on the anatomical image obtained from x-ray, and separating the components parallel to the pathway—namely, the intracellular axonal currents—from those perpendicular to it—namely, the volume currents flowing in and out of the pathway, for display as waveforms, is extremely powerful tool for diagnosing neural dysfunction sites (Figure 4B) because the inward current is particularly affected at neural dysfunction sites.

### 3.2 Artifact reduction

In spinal cord and peripheral nerve biomagnetic measurements, electrical stimulation is applied to the spinal cord or peripheral nerves, and the induced magnetic fields are detected. When the site of stimulation is close to the observation area, or when the stimulation intensity is high, electrical stimulation artifacts may overlay on the target signal waveform, making magnetic source analysis difficult. The concept of spatial filtering is utilized not only for magnetic source analysis but also for the removal of such artifacts associated with electrical stimulation. We currently apply two types of artifact reduction algorithms, which called dual signal subspace projection (DSSP) (30) and common-mode subspace projection (CSP) (31). Both algorithms aim to reduce artifacts by projecting into a subspace with the signal space where the electrical stimulation artifacts are minimized.

DSSP utilizes the positional information of each SQUID sensor to separate the signal waveforms that originate directly above the sensor array from those that originate from other areas. It then removes the signals that come from areas other than directly above the sensor array. In many cases, the site of electrical stimulation is outside the sensor array, making this artifact reduction effective.

However, when the site of electrical stimulation is close to the sensor array, such as when stimulating a finger and measuring the magnetic field at the wrist for neural function imaging of carpal tunnel area as described in Section 5.4, DSSP cannot separate the artifacts. In such cases, an effective strategy involves distancing the wrist from the sensor array using an appropriate spacer to isolate and measure only the electrical stimulation artifacts. This is done separately from the magnetic field measurements of the wrist. Subsequently, one identifies a shared subspace between the signal space that contains both the original target signal and the electrical stimulation artifacts, and another signal space that contains only the electrical stimulation artifacts. This shared subspace is then separated out. This approach is referred to as CSP.

With the advent of these artifact-reduction algorithms, it has become possible to apply supramaximal electrical stimulation and to stimulate near the observation area, dramatically improving the signal-to-noise ratio of the measurements. This has expanded the applicability of the magnetoneurograph system to include areas beyond the spinal cord.

## 4 SQUID system for magnetoneurography

**Figure 5:**
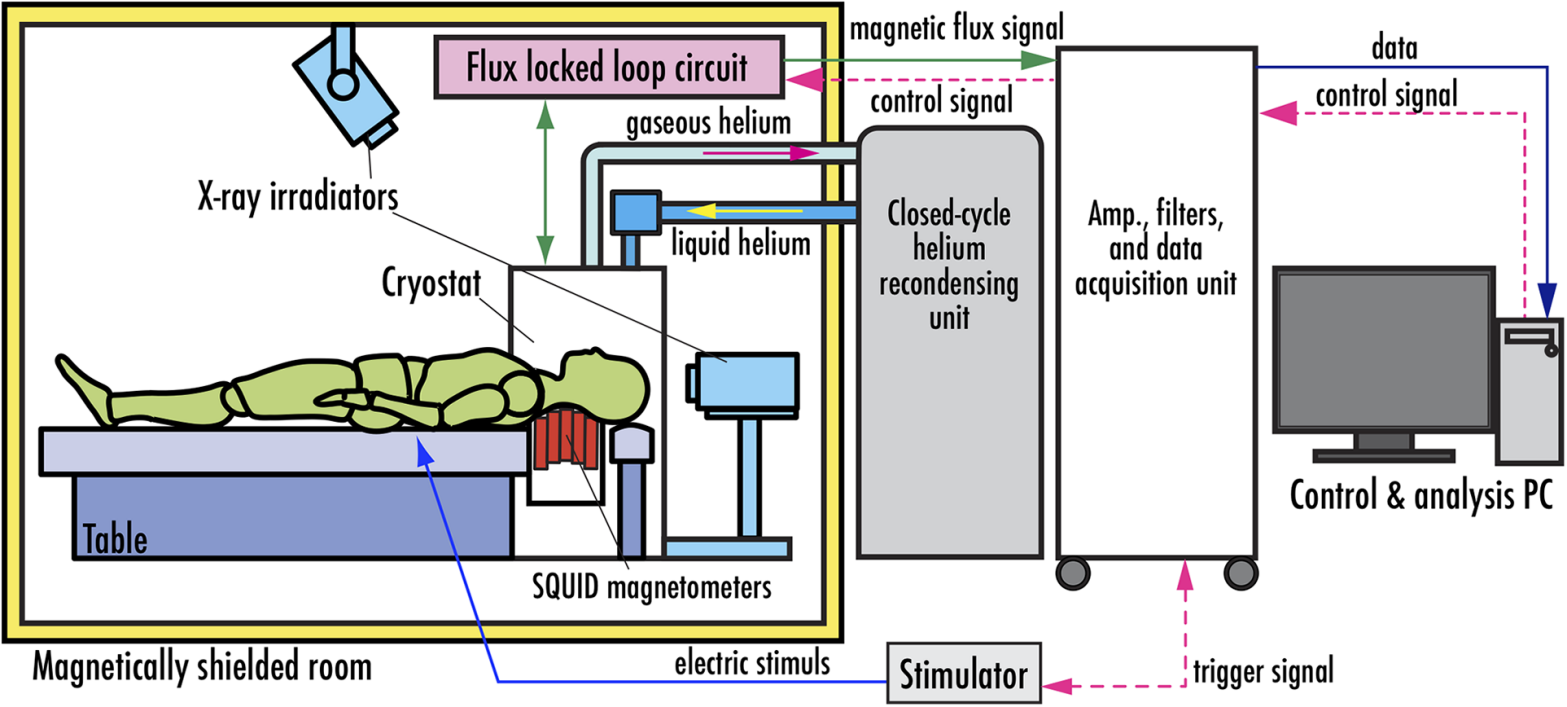
System configuration of SQUID magnetoneurograph system. Excerpt from reference (29).

Figure 5 shows the system configuration of the magnetoneurograph system (29). Fundamentally, it is composed of an array of SQUID magnetometers, an ultra-low temperature container, called cryostat or Dewar vessel, to maintain the SQUIDs in a superconducting state, FLL circuits, and a data acquisition unit to digitally record signals from the FLL. While this architecture shares similarities with conventional biomagnetic measurement systems, it has distinctive features particularly in the SQUID magnetometers and cryostat. Measurements are conducted within a magnetically shielded room to eliminate external magnetic fields. Additionally, the system is designed to allow for both frontal and lateral x-ray imaging in the same posture as during the measurement. This facilitates the acquisition of anatomical images, which are useful for aligning the sensors with the subject and providing supplementary information for further magnetic source analysis.

**Figure 6:**
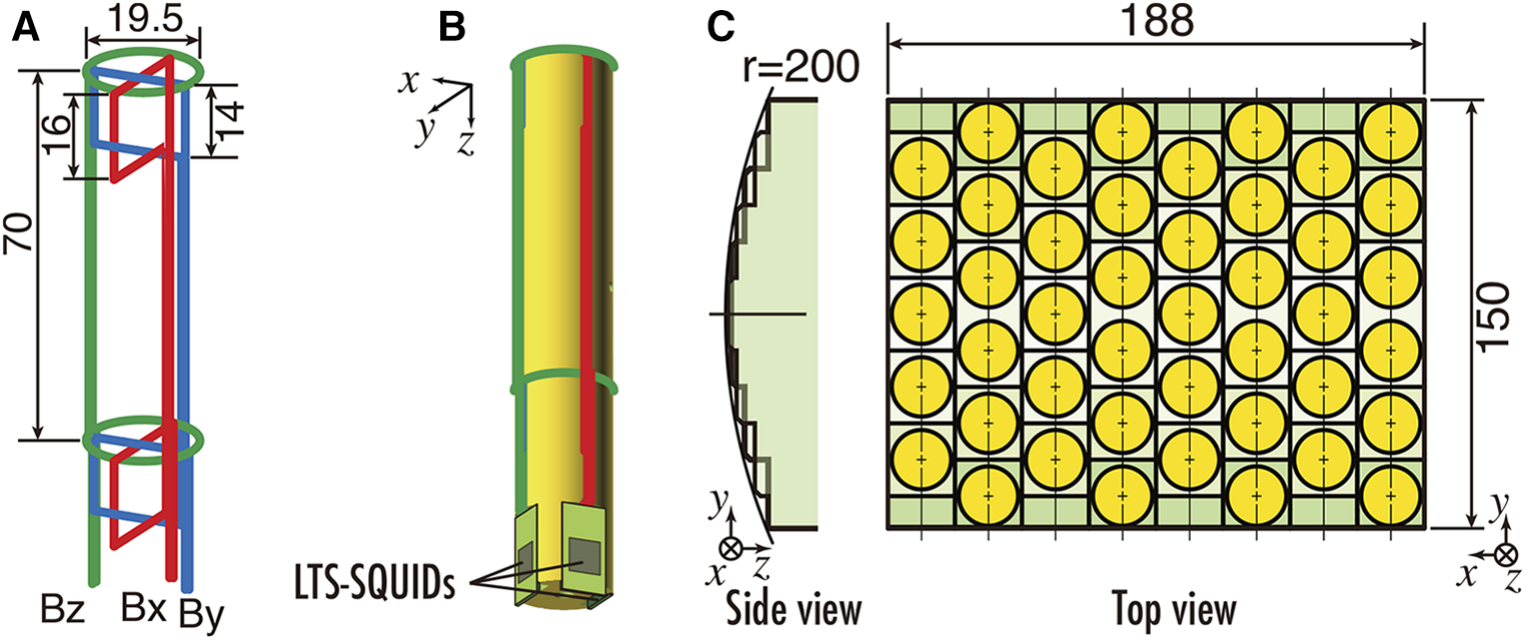
(**A**) Configuration of pickup coils of vector SQUID sensor. (**B**) Appearance of vector SQUID sensor. (**C**) Sensor arrangement of SQUID magnetoneurograph system. All units are in mm. Excerpt from reference (29).

In the specific anatomical regions of interest, such as the cervical spinal cord or carpal tunnel peripheral nerves, the spatial extent of the magnetic field observation area is considerably smaller in comparison to conventional applications like MEG or MCG. To maximize the extraction of magnetic field data from these concerned regions, we apply “vector-type” SQUID magnetometers, as shown in Figures 6A,B. This configuration comprises a singular sensor module, outfitted with three orthogonally oriented differential pick up coils, which are a single axial-type and two planar-type, each coupled with a discrete SQUID. This arrangement affords the simultaneous detection of magnetic field components along three orthogonal axes. Consequently, this not only enables the conventional assessment of magnetic field components normal to the body surface but also permits the measurement of tangential components. The diameter of the cylindrical bobbin equivalent to the diameter of the axial-type pick up coil is approximately 20 mm. The dimension of the two planar-type pick up coils are 16 × 19.5 mm^2^ and 14 × 19.5 mm^2^, respectively. The system achieves a magnetic field resolution of less than 2 fT/Hz^0.5^ within the white noise region.

The sensor array mounted along the upper surface of the cryostat protruding section is equipped with vector-type SQUID magnetometers at 44 locations over an area of 188 mm × 150 mm, resulting in a total of 132 channels, as shown in Figure 6C. Furthermore, the sensor array is designed to conform to the dorsal surface of the neck, with sensors arranged along a gently curved cylindrical surface when viewed from the side. The inter-sensor spacing is 23.5 mm in the X-direction and 25.5 mm in the Y-direction.

The output of the SQUID magnetometers is interfaced with the previously mentioned FLL, and the subsequent output from the FLL undergoes analog filter and amplification before digital data acquisition. The conduction velocity of neural signals is estimated based on the difference in peak latency of the signal waveforms detected by adjacent magnetometers and the positions of these magnetometers. Therefore, it is crucial to accurately obtain the difference in peak latency, that is, the phase difference between signal waveforms across different magnetometers. This velocity typically ranges from 60 to 100 m/s. Given that the magnetometers are spaced approximately 25 mm interval, a sampling rate on the order of tens of kHz is required to accurately capture the phase differences. Despite the fact that the bandwidth of magnetic field signals from the spinal cord and peripheral nerves is a mere few kHz, the system is capable of digital sampling at a relatively high frequency, up to 40 kHz, to meet this requirement.

**Figure 7:**
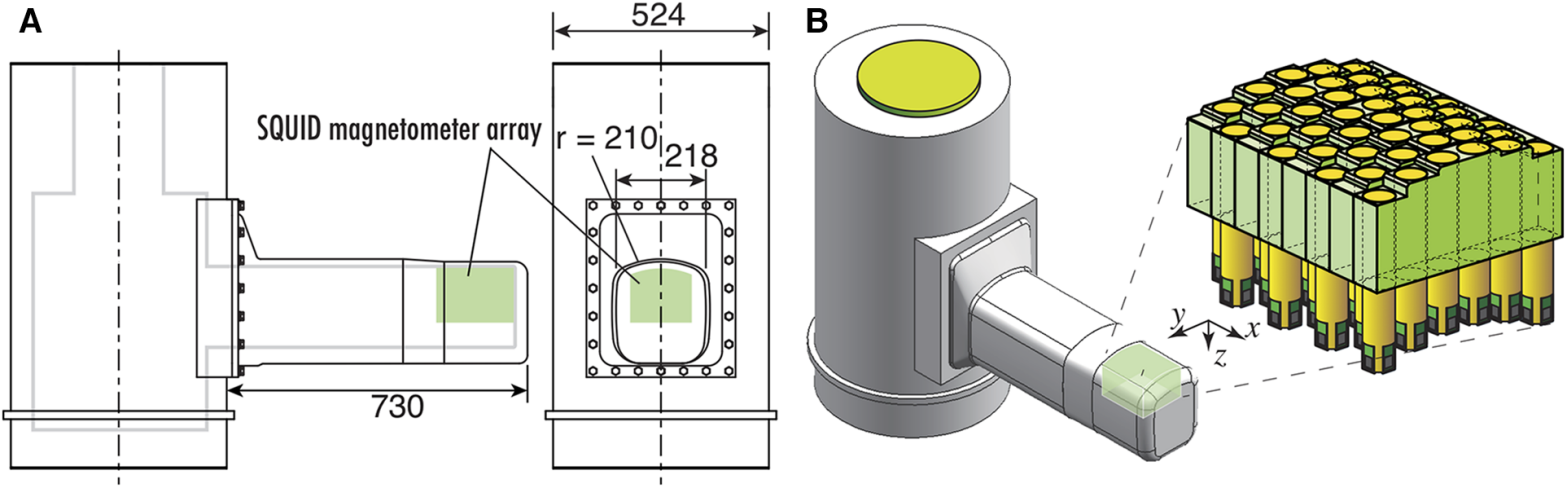
(**A**) Cross sectional image with dimensions of cryostat for SQUID magnetoneurograph system. (**B**) Appearance of cryostat and position and orientation of sensor array implemented to cryostat. Excerpt from reference (29).

The cryostat for maintaining the superconducting state of SQUIDs features a double-walled structure with vacuum layer for thermal insulation and is constructed from glass fiber-reinforced plastic avoid magnetic interference. Figure 7A shows the cross-sectional schematic and external appearance of the cryostat. The cryostat has a unique design that allows for sensor implementation to protrude horizontally from the side surface of the cylindrical main body to reserve liquid helium, enabling close proximity to the subject's dorsal body surface in a supine position, as shown in Figure 7B. The sensor array is mounted along the upper surface of this protruding section. Subjects can lie supine on a bed and bring their neck or waist into close contact with this section for measurement. The distance from the outer surface of the observation area to the cryogenic layer is approximately 12–14 mm, which is shorter than in conventional MEG or MCG systems, thereby allowing the SQUID magnetometers to be placed as close as possible to the magnetic source. The liquid helium reservoir has a capacity of approximately 92 L and can sustain the superconducting state of the SQUIDs by refilling liquid helium once a week.

This cryostat structure for supine subjects was initially developed in 2007, and has undergone multiple extensions and improvements of its protruding section as the application range of magnetoneurography has expanded. The initial prototype of the supine-position cryostat had a protruding section with a length of 390 mm and a width of 170 mm, targeting only the cervical spinal cord (14). The length was later extended to approximately 530 mm to include the lumbar spine as a measurement target (15). In the latest system developed in 2020, the length has been further extended by 200 mm–730 mm to accommodate larger subjects and to facilitate measurements of induced postural peripheral nerve magnetic fields. And the width was extended to 218 mm to hold the larger sensor array.

## 5 Applications of magnetoneurography

### 5.1 Procedure for magnetoneurography

Thanks to the uniquely-shaped cryostat and the sensor array oriented upward, the magnetoneuro- or magnetospino-graphic signals can be collected from any part of the body by simply placing it above the sensor array. Additionally, the signals processing algorithms based on spatial filtering techniques, DSSP and CSP, can be applied to reduce artifacts originating from electric stimulation and the target signals hidden by artifacts are extracted. These artifact reduction algorithms enable to apply supramaximal stimulation or stimulation nearby the observation area for obtaining the better signal-to-noise ratio. It was drastically effective to expand the application range of the magnetoneuro- and magnetospino-graphy. In this section, the recent achievements of the applications of magnetoneuro- and magnetospino-graphy are reviewed.

**Figure 8:**
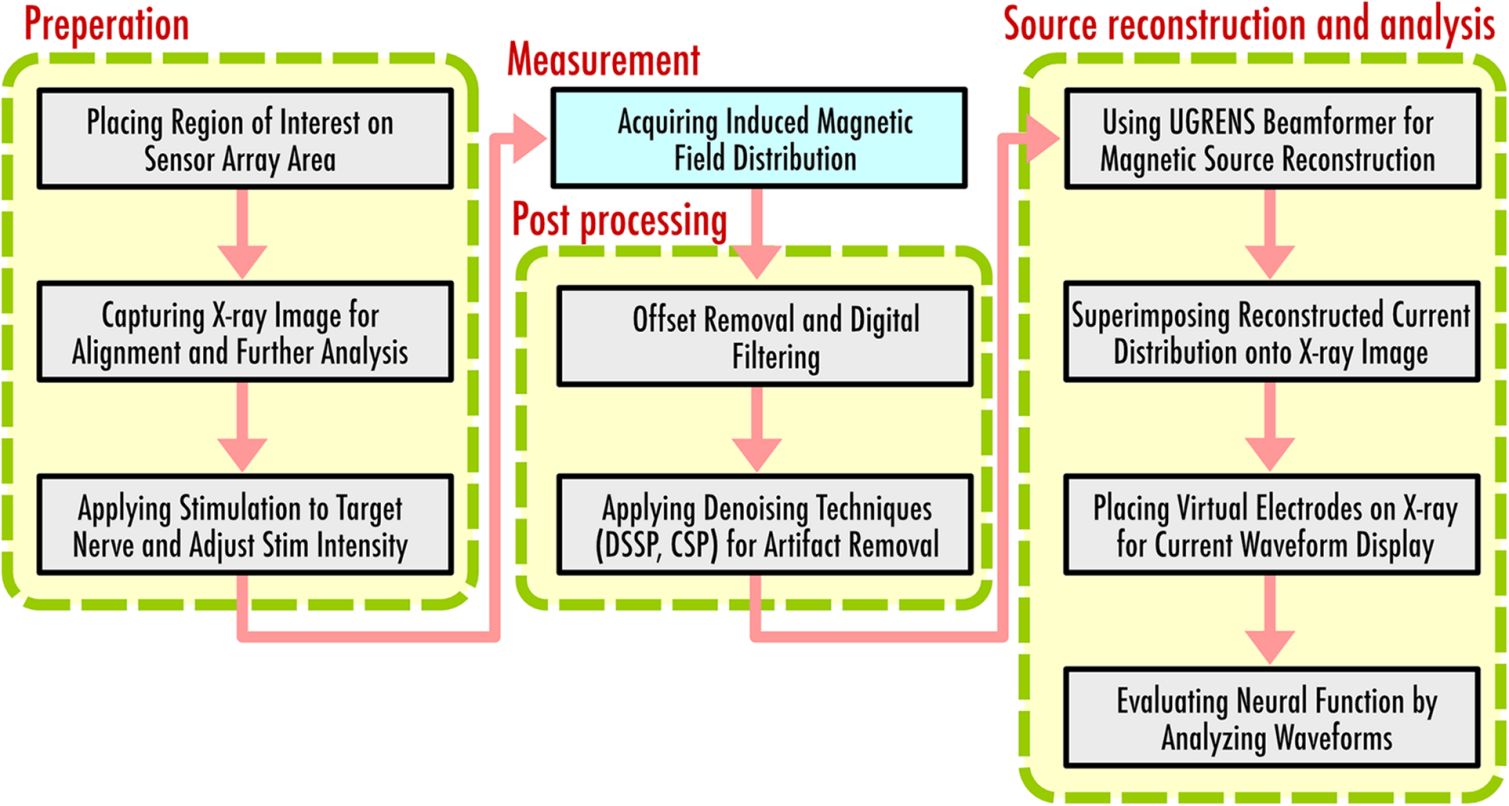
Flow diagram to indicate procedure of magnetoneurography.

The currently established procedure for magnetoneurography comprises several steps designed to ensure accurate data acquisition and analysis. Figure 8 shows a flow diagram to indicate the procedure of magnetoneurography. Initially, the anatomical region of interest is brought into close contact with the upper surface of the sensor array, followed by the capturing of x-ray images to ascertain the alignment. This x-ray serves a dual purpose, as it is also utilized as supplemental information for subsequent magnetic source analysis.

Following the alignment, electrical stimulation is applied to the target nerve, and the resulting induced magnetic field distribution is acquired. During the measurement, simultaneous electrical potential tests may be conducted to confirm that the stimulation is applied appropriately. The same stimulation is repeated hundreds to thousands of times, and additive averaging is applied to improve the signal-to-noise ratio. Post averaging, denoising techniques such as DSSP or CSP are applied to reduce the stimulation artifacts overlapping in the target signal. Spatial filtering technique for magnetic source analysis, known as UGRENS beamformer, is then applied to the artifact-reduced magnetic field data to obtain the reconstructed current distribution within the region of interest determined based on the previously captureds x-ray image.

The reconstructed current distribution is superimposed onto the x-ray image, providing comprehensive visual presentation. Virtual electrodes are placed on the x-ray image, enabling the waveform display of temporal changes in currents at specified locations and orientations. In many cases, these virtual electrodes are aligned along predetermined neural pathways based on the x-ray imaging. The current components both parallel and perpendicular to these neural pathways are then individually displayed and evaluated. Assessments of neural function are made possible by carefully observing the reconstructed current waveforms examining the shifts of peak latency and amplitude between adjacent electrodes. Conduction velocity of neural signals is also estimated from the peak latency and the intervals of virtual electrodes. Local anomaly of the conduction velocity serves as a significant information for diagnosing the site of neural impairment.

### 5.2 Measurement for cervical spinal cord by peripheral nerve stimulation

The cervical spinal cord was the initial target of our measurements, and it has the most comprehensive data accumulated to date. Two stimulation methods exist for acquiring induced signals: one involves electrical stimulation of the thoracic spinal cord, while the other pertains to transcutaneous electrical stimulation of peripheral nerves at the elbow or wrist. Thoracic spinal cord stimulation yields relatively large signals and features a straightforward neural pathway that simplifies analysis. The results obtained by thoracic spinal cord stimulation had also been confirmed to align with those from evoked potential measurements in spinal canal conducted via catheter electrode, and this had served to initially validate the appropriateness of spinal cord functional assessments through magnetoneurography for patients (32). However, this method necessitates the insertion of a catheter electrode into the spinal canal, thereby restricting its application to a limited group of subjects, such as patients awaiting cervical spine surgery. On the other hand, transcutaneous electrical stimulation of peripheral nerves imposes no such limitations on subject selection and can be applied to preventive screenings or prognostic evaluations. In the initial stages of the research, peripheral nerve stimulation yielded small signals and the complex transition pattern of the magnetic field distribution, making the interpretation of the measurement data challenging. However, the development of spatial filtering methods for magnetic source analysis has enabled high spatial resolution assessment of the action currents converging from the upper limb peripheral nerves to the spinal cord, paving the way for its application in functional diagnostics.

A nerve comprises multiple neural fibers of varying diameters. The conduction velocity of each fiber is diameter-dependent, leading to dispersion in the waveform and attenuation in the intensity of the neural signal as it travels away from the stimulation site. Consequently, applying a larger stimulation closer to the observation area yields larger evoked magnetic field signals and higher signal-to-noise ratios. However, if the electrical stimulation site is proximal to the sensor array, or if the intensity of the stimulation is high, electrical artifacts may contaminate the signal waveform. These artifacts often preclude further magnetic source analysis until they are mitigated through the artifact reduction such as DSSP or CSP.

**Figure 9:**
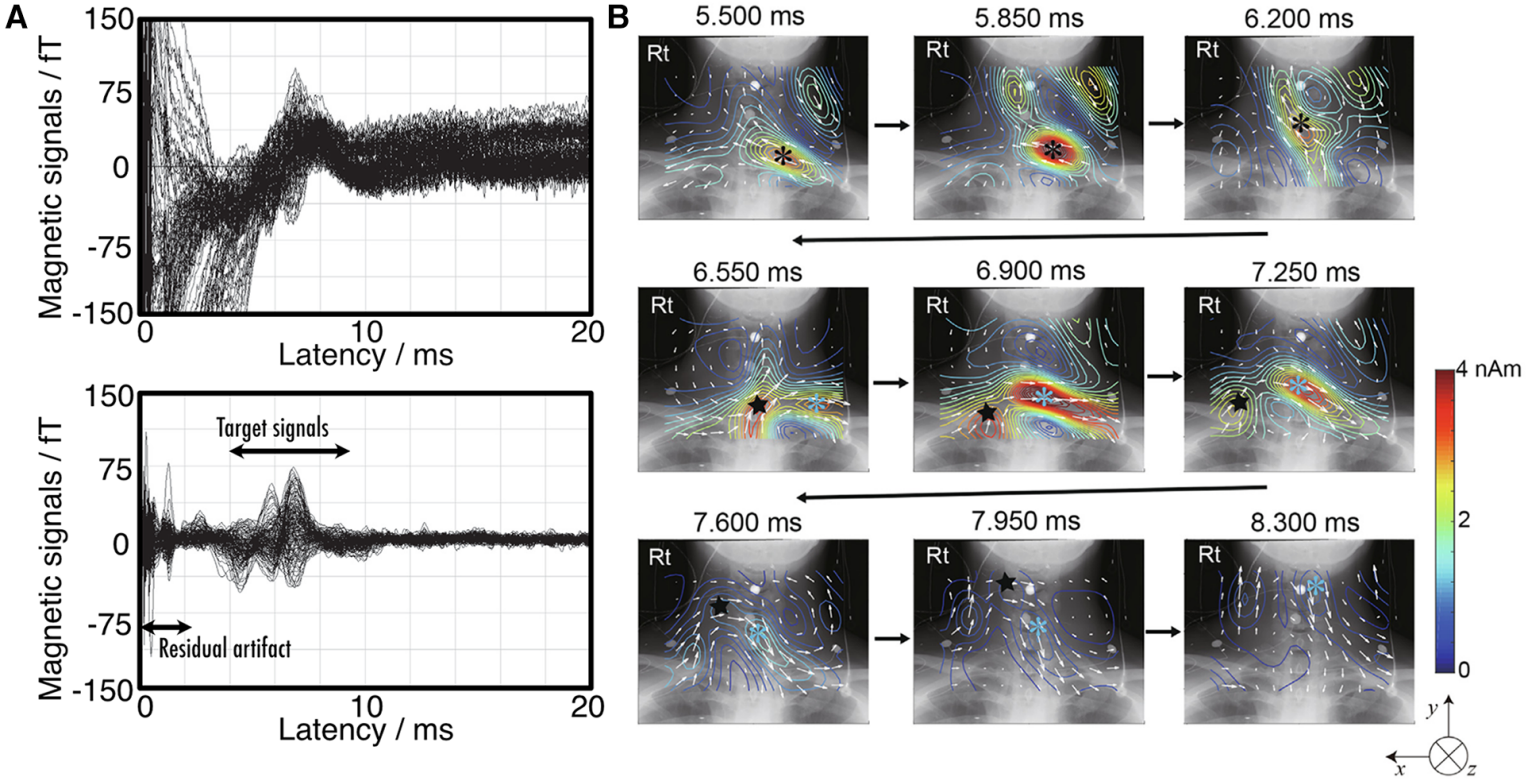
Example of magnetoneurography for cervical spinal cord induced by ulnar nerve stimulation at elbow. (**A**) Waveforms of magnetic field signals obtained from SQUID sensors before applying artifact reduction (upper) and after removing the artifact by DSSP (lower). (**B**) Current distribution around neck reconstructed by UGRENS beamformer from artifact-reduced magnetic field data. On pseudo color map, black and blue asterisks represent positions of leading and trailing currents, respectively. Black star indicates position of inward current. Magnetic field measurement was conducted with supramaximal stimulation given to ulnar nerve at elbow and magnetic fields were captured at back of neck. Evoked magnetic field signals were digitally recorded at 10,000 Hz of sampling rate after applying a band pass filter of 100–5,000 Hz, and then averaged until signal-to-noise ratio exceeded about 5 or higher (4,000–8,000 times). These figures are excerpts from reference (33) with original data directly provided by authors.

Figure 9A shows an example of magnetic fields recorded in the cervical spine of a healthy subject, induced by electrical stimulation of the ulnar nerve at the elbow (33). While the signal obtained is approximately twice as large as that elicited by wrist ulnar nerve stimulation, the waveform is contaminated by electrical stimulation artifacts as shown in the upper plots in (a). These artifacts overlays on the target signals appearing at 5.5 ms in latency, complicating the application of magnetic field analysis. The lower plots in Figure 8A shows the result after the application of DSSP, where the artifacts have been significantly reduced, allowing for clear identification of the response waveform. Figure 9B shows the transition of current distribution reconstructed by UGRENS beamformer from artifact-reduced magnetic field data.

It is anatomically known that the median and ulnar nerves, two peripheral nerves of the upper limb, pass through intervertebral foramina at different spinal levels before merging into the spinal cord. When compared to the case where a magnetic field signal is elicited by stimulating the median nerve (32), it becomes evident from the transition of the reconstructed current distribution that these neural pathways can be distinctly differentiated and evaluated separately. Thus, thanks to the improvement in the signal-to-noise ratio by elbow stimulation, we have gained the resolution necessary to investigate and distinguish between the two nerves.

### 5.3 Lumbar and thoracic spinal cord

The lumbar region was the next area to be targeted for magnetoneurography, following measurements in the cervical spinal cord. Due to the greater distance from the body surface to the magnetic field source compared to the cervical spinal cord, signal detection was initially challenging. However, after improvements in sensor array and cryostat, it became possible to stably detect signals from cauda equina or lumbar spinal canal induced by stimulation of the peripheral nerves in the lower limbs (34, 35). Nonetheless, for the thoracic spinal cord there is no suitable nerves for transcutaneous stimulation. Unlike the lumbar region, measurements of the signal from thoracic spinal cord were still challenging in the case of lower peripheral nerve stimulation, due to the considerable distance between the stimulation site and the observation area, which led to signal attenuation.

For this reason, a stimulation method called “synchronized bilateral sciatic nerve stimulation” was devised (36). First, the time it takes for neural signals induced by stimulating the sciatic nerves in both popliteal fossae to reach the cauda equina is pre-measured individually. Then, both sciatic nerves are sequentially stimulated with this time difference taken into account. As a result, in neural pathways above the cauda equina, the neural signals that have propagated through the left and right sciatic nerves converge in-phase, leading to a signal strength that is the sum of the signals generated when each sciatic nerve is stimulated individually. Utilizing this stimulation method, it was enabled to induce larger signals and observe signal propagation in the thoracic spinal cord with an acceptable signal-to-noise ratio.

**Figure 10:**
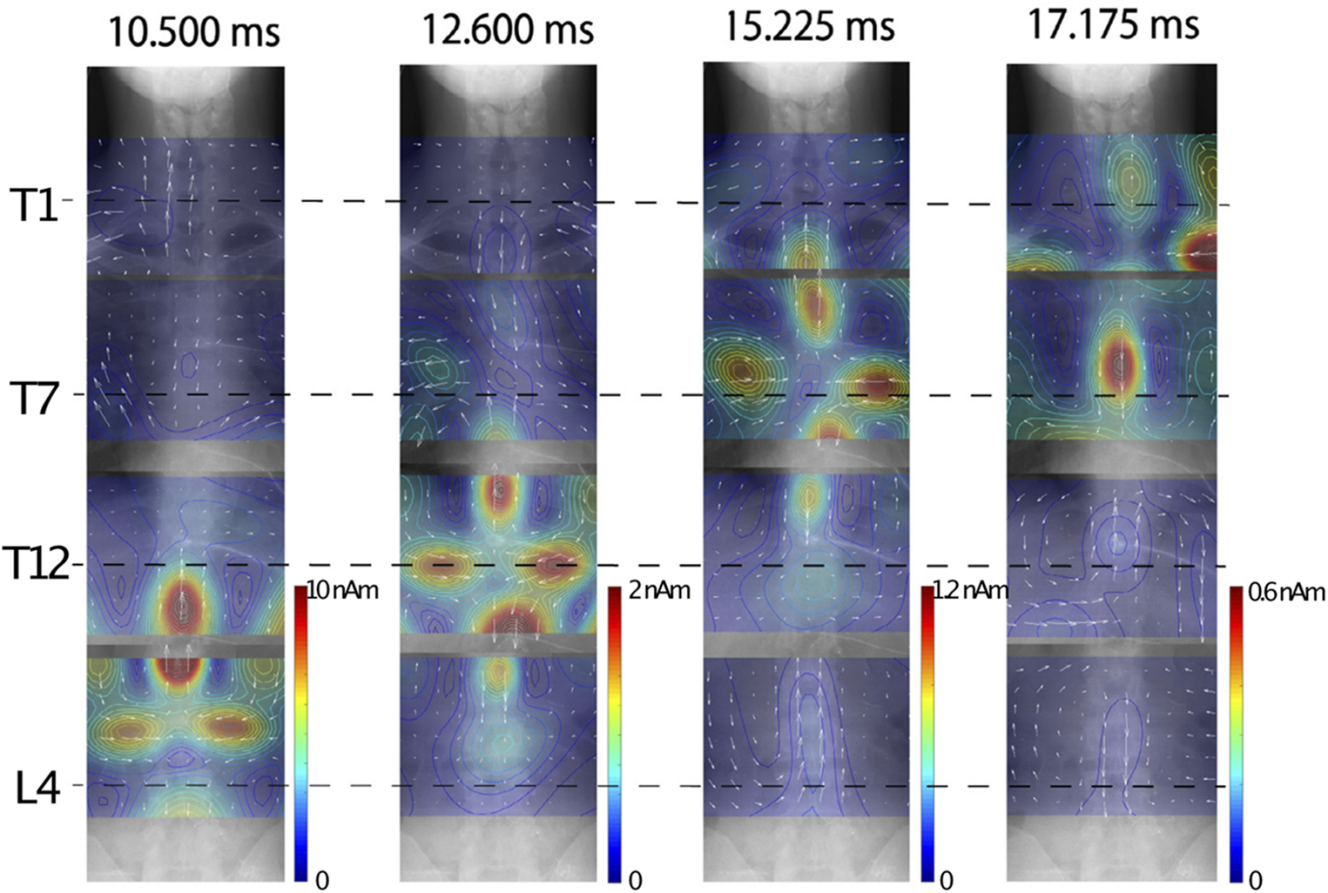
Example of chronological change of reconstructed current distribution along thoracic spinal cord induced by synchronized bilateral sciatic nerve stimulation. Intensity of stimulation was set to a supramaximal level. Induced magnetic field was captured from subject without neurological symptoms by shifting measurement areas four times. Sampling rate and band-pass filter were set to 40,000 Hz and 100–5,000 Hz, respectively. Evoked magnetic field data was averaged 2,000 times in lumbar area, 4,000 times in lower thoracic area, and 8,000 times in middle to upper thoracic areas. Stimulation artifacts were reduced by DSSP, and then UGRENS beamformer was applied to artifact-reduced data to reconstruct current distribution. These figures are excerpts from reference (36) with original data directly provided by authors.

Figure 10 shows an example of chronological change of reconstructed current distribution along the the thoracic spinal cord, induced by synchronized bilateral sciatic nerve stimulation. Magnetic field measurements were conducted in four segments: the T1, T5, T12 levels and the lumbar region, and then the reconstructed current distributions from each measurement superimposed onto full-body x-ray images for display. The intra-axonal current components comprised of leading current and trailing current along with the inward current component situated between them could be observed from the lumbar region up to the T1 level.

Owing to introducing the synchronized bilateral sciatic nerve stimulation, examinations of the entire spinal cord and spinal nerves from the cauda equina to the cervical cord have now become possible, where previously the assessment of the thoracic spinal cord was challenging.

### 5.4 Magnetoneurographic measurements for peripheral nerves

Magnetoneurography is also applicable for the functional imaging of peripheral nerves. Induced magnetic fields measurements and current reconstructions have been reported for the carpal tunnel (37, 38), elbow (39), upper arm (40), and brachial plexus (41, 42), based on peripheral nerve stimulation at the digital nerves, median and/or ulnar nerves at the wrist or elbow. In each case, the reconstructed current waveforms obtained from the measured magnetic field data were compared with the results of conventional surface potential measurements. The coincidence between the peak latency of the inward current at the virtual electrodes placed along the nerve pathways and the peak latency of nearby surface potential waveforms has been confirmed. This suggests that the peak of the inward current indicates the location of axonal depolarization at that moment. Examinations targeting patients with carpal tunnel syndrome (43) and cubital tunnel syndrome (37) have also been reported. Particularly for carpal tunnel syndrome, data have been obtained suggesting that injury sites undetectable by conventional surface potential measurements due to a thick transverse ligament can be identified using magnetoneurography thanks to its higher spatial resolution.

### 5.5 Dorsal horn postsynaptic activity

**Figure 11:**
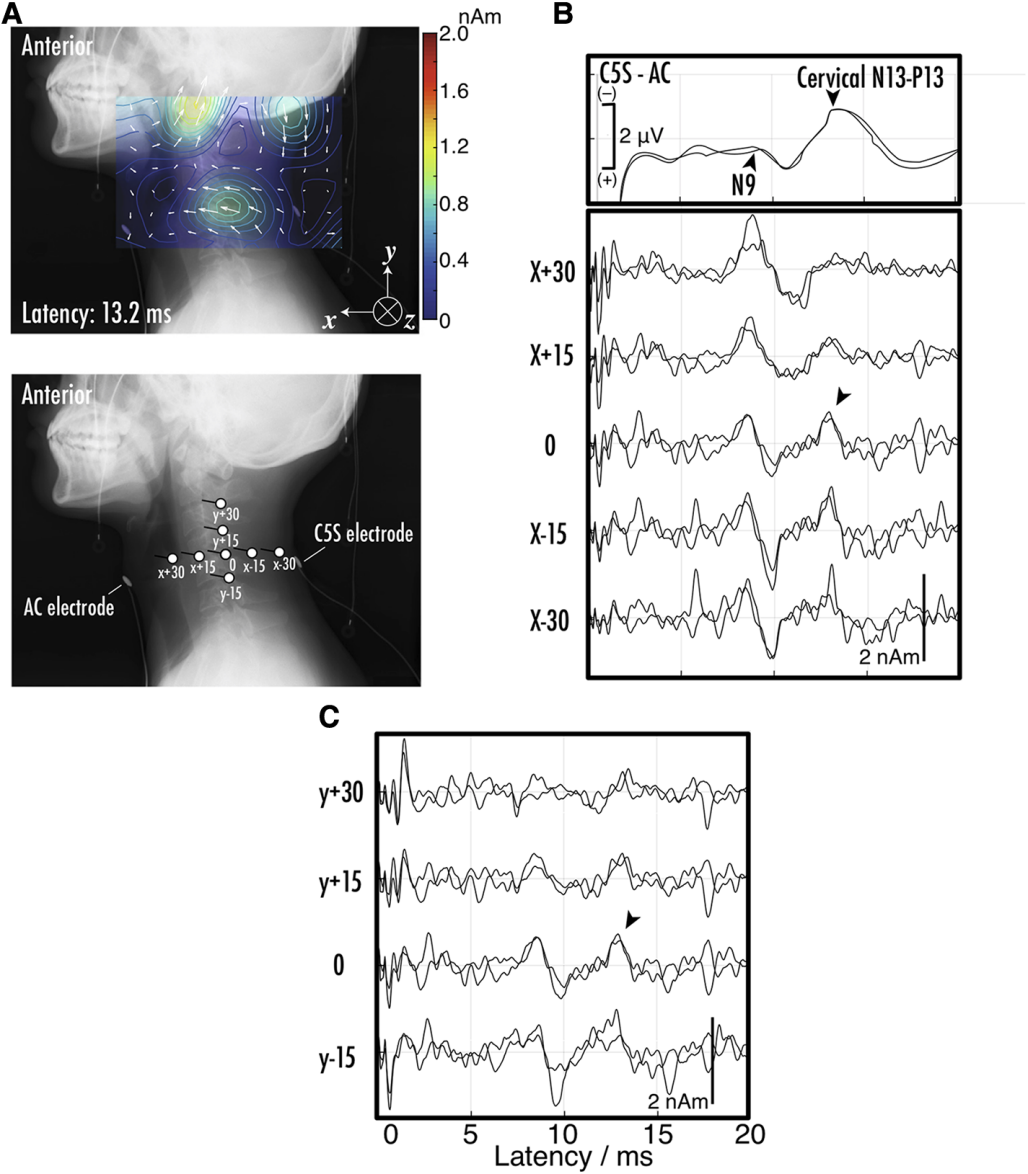
(**A**) Example of reconstructed current distribution 13.2 ms after stimulation to median nerve at wrist overlaid on lateral x-ray image. Evoked magnetic field in response to supramaximal median nerve stimulation at wrist was captured from right side of neck. Sampling rate and band-pass filter were set to 40,000 Hz and 10–5,000 Hz, respectively. After digitization, magnetic field data was averaged 1,000–2,000 times to improve signal-to-noise ratio. Stimulation artifact was reduced by DSSP, and then RENS beamformer was applied to artifact-reduced data to reconstruct current distribution. Two measurements under same conditions were conducted to verify reproducibility. (**B**) The positions and orientations of virtual electrodes to indicate waveforms of reconstructed current at specific sites. Anterior cervical (AC) and C5S posterior electrodes were also shown. Labels such as X-30 are referred in (**C**) upper waveforms indicate somatosensory evoked potentials (SEPs) recorded at C5S-AC montage. Middle and bottom sets of waveforms represent reconstructed current on C5 level spinal canal. Labels such as X-30 are corresponding to those in (**B**) the spikes between 0 and 2 ms in latency are considered as residual stimulus artifacts. These figures are excerpts from reference (44) with original data directly provided by authors.

Magnetoneurography is not only capable of observing nerve signals propagating along axons, but also allows for the observation of synaptic activity. Figure 11 shows an example of the magnetoneurogram at the cervical spinal cord specifically measured from the lateral side of the neck with stimulation of the median nerve at the wrist (44). When compared with the waveforms of the surface potential between the dorsal and ventral sides at the C5 level simultaneously recorded with the magnetic field, a peak could be confirmed in the reconstructed current waveform at the same latency as the potential component called the N13–P13, which appears about 13 ms after the median nerve stimulation at the wrist. From the reconstructed current distribution map superimposed on the lateral x-ray image at that latency, a clear current component was confirmed flowing from the dorsal to the ventral direction at the C5 level. This is consistent with the N13–P13 component corresponding to postsynaptic activity in the dorsal horn. Conventional magnetic field measurements from the dorsal side have made it difficult to detect magnetic fields originating from currents flowing in the dorsal-ventral orientation. However, such detection has become possible by measuring the magnetic field from the lateral side of subjects in the side lying position. These results suggest that magnetoneurography not only paves the way for the examination of conductive disorders in the dorsal column but also opens up the possibility of evaluating functions of the dorsal horn, which was challenging with traditional surface potential measurements.

### 5.6 Investigating origin of P9 component

**Figure 12:**
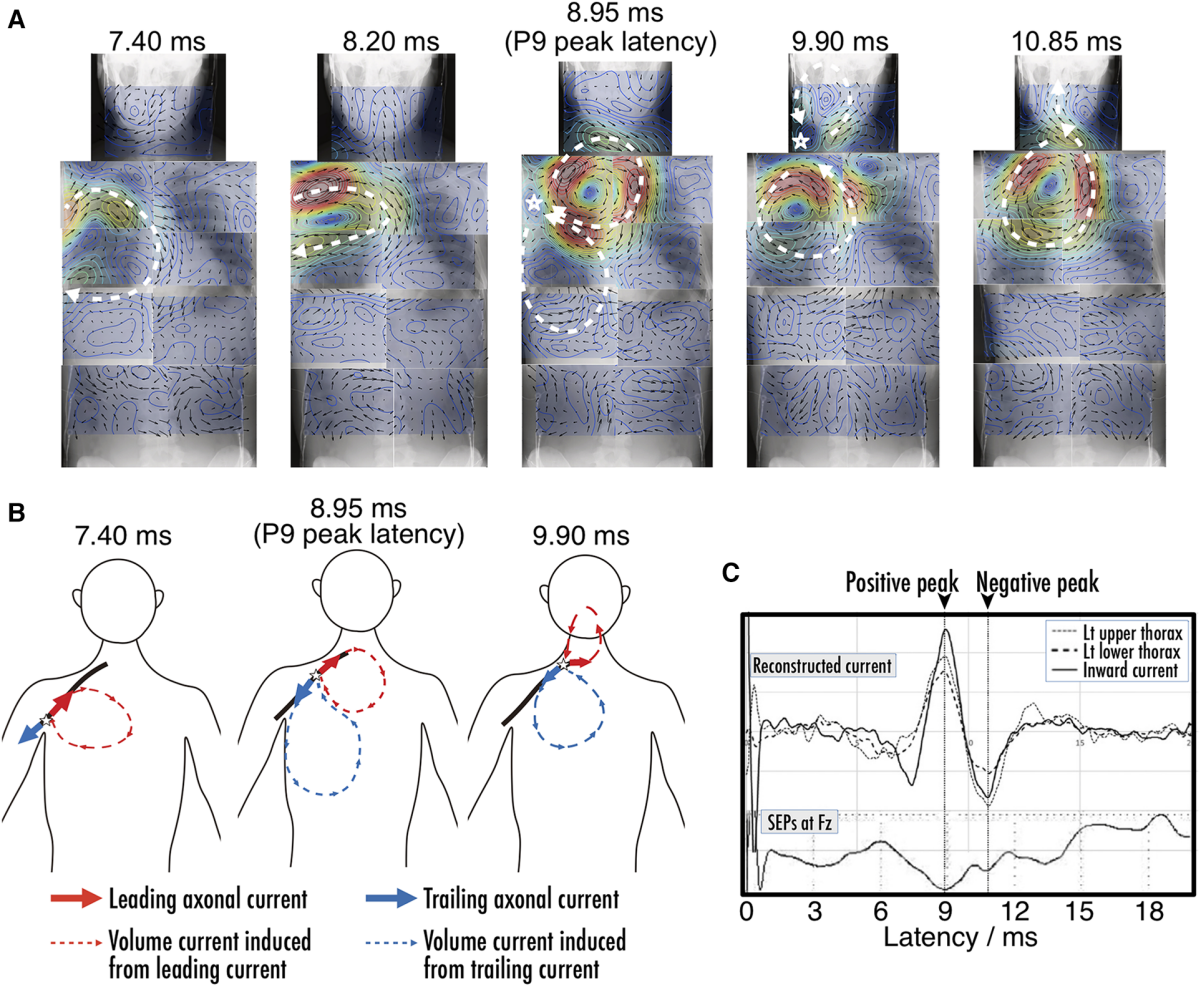
(**A**) Chronological change of reconstructed current distribution over neck and trunk region before and after P9 component. Magnetic field, which served as basis for reconstructed current distribution evoked by median nerve stimulation at wrist, was digitally recorded and averaged 4,000 times. Measurement area was divided into nine sections as follows: neck, bilateral upper thorax, bilateral lower thorax, bilateral upper abdomen, and bilateral lower abdomen, and reconstructed current distribution from each section were superimposed on whole body x-ray image. (**B**) Illustration of dynamics of current distribution before and after P9 component. Red and blue components indicate leading intra-axonal current and associated volume current, and trailing and associated volume currents, respectively. (**C**) Waveforms of reconstructed volume currents from virtual electrodes placed at upper thorax, lower thorax, and between them to detect inflow into axon along with SEP obtained at Fz. Positive peak latencies of current intensity were coincident with each point, and also corresponded with P9. The spikes between 0 and 2 ms in latency are considered as residual stimulus artifacts. These figures are excerpts from reference (45) with original data directly provided by authors.

Magnetoneurography serves not only as a diagnostic tool for identifying neurological disorders through the examination of nerve function, but also as a valuable source gaining new insights into electrophysiology. Figure 12 shows an example of the reconstructed current distribution aimed at elucidating the origin of the component commonly known as the P9 component in traditional somatosensory evoked potential tests, which appears around a latency of 9 ms in the brain (45). This distribution was based on the magnetic field induced by stimulating the median nerve at the wrist, and the magnetic field was captured over a wide area extending from the brachial plexus to the chest. This example combines results from nine separate measurement areas. Even when data from multiple separate measurements are combined over a wide range, stable and reliable results were obtained through magnetoneurography, which is less susceptible to unstable factors such as electrode contact resistance. P9 is one of surface potential component called far field potential that occurs at a distance from its source. While it has been known that variation of volume currents is involved, there has been controversy over whether the signal propagating along the peripheral nerves of the upper arm originates when entering the thorax from the upper arm or when entering the neck from the thorax. The results from Figure 12 suggest the latter is correct. Such differentiation was not feasible with traditional surface potential measurements, but became possible through magnetoneurography, which allows for the visualization of volume current variations with relatively high spatial resolution.

## 6 Comparison with other modalities

### 6.1 Spatial and temporal resolution

**Figure 13:**
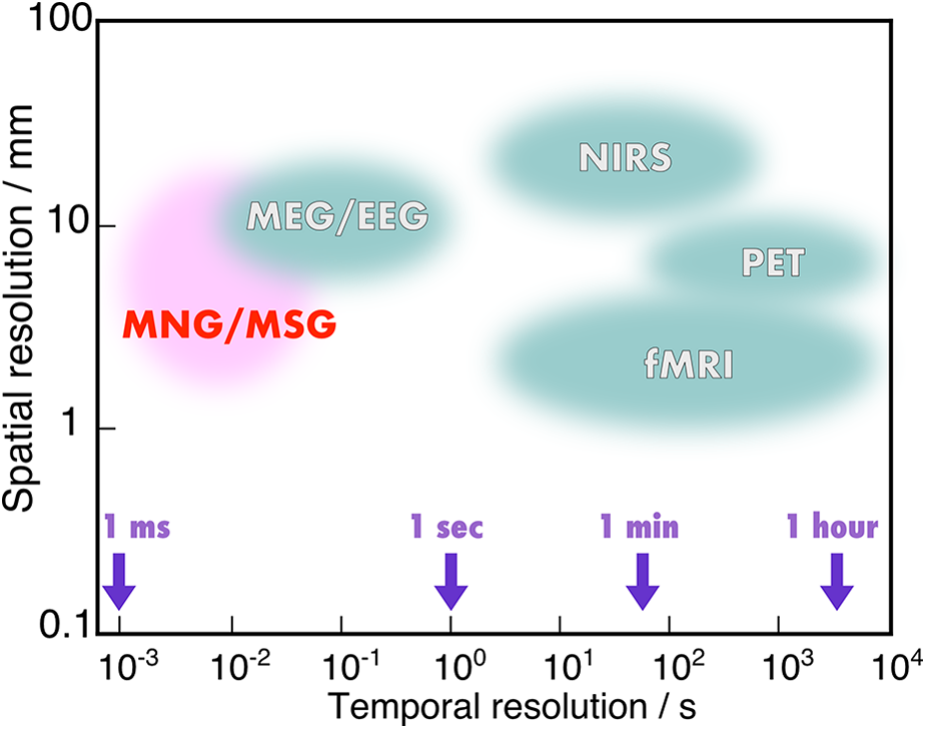
Spatial resolution and temporal resolution of noninvasive neural functional imaging modalities. The values for MEG/EEG, NIRS, PET, and fMRI were cited from reference (46).

In the observation of neural functions in the spinal cord and peripheral nerves, other than brain, traditional methods have been limited to electrophysiological signal detection. However, when considering brain neurons, various non-invasive modalities become available. These can be broadly categorized into two types: those that measure neural electric currents generated by neural activity, and those that measure local cerebral blood flow changes. The former category includes electroencephalography (EEG) and MEG, with magnetospinography also falling into this classification. The latter category comprises functional magnetic resonance imaging (fMRI), positron emission tomography (PET), and near infra-red spectroscopy (NIRS). Modalities in the first category directly observe the electrical activity of neurons and thus possess high temporal resolution. In contrast, the second category focuses on observing changes in cerebral blood flow, which fluctuates with the consumption of oxygen and chemical energy associated with neural activity. Due to the involvement of chemical reactions, these methods find it difficult to capture activities requiring high temporal resolution, such as the propagation of neural signals along axons. The spatial and temporal resolutions of these modalities are illustrated in Figure 13.

### 6.2 Comparison with magnetoencephalography

While the features of magnetoneurography in contrast to traditional MEG have already been addressed in the previous sections several times, this section specifically focuses on elaborating the aspects of spatial resolution and magnetic source analysis. The spatial resolution in biomagnetic field measurements is difficult to evaluate as a definitive value, as it depends on sensor array density, signal strength, and the distance of the magnetic source. For nerves located in relatively superficial areas like the elbow and where the number of stimulated nerve fibers is large, the signal intensity is larger than that of MEG and a good signal-to-noise ratio allows for millimeter-order resolution. However, when targeting the spinal cord, where the magnetic source is located deep from the body surface, the signal is considerably weaker compared to MEG, and achieving the same level of spatial resolution can often be challenging. In magnetoneurography, however, as well as the evoked response measurements utilized in MEG, the signal-to-noise ratio can be improved by repeatedly providing the same stimulus and averaging the responses. Particularly in the case of magnetoneurography, unlike MEG, there is hardly any habituation to the stimulus, resulting in very high reproducibility of responses to the same stimulus. Notably, even after averaging thousands of responses, a count far exceeding the typical number of repetitions in MEG measurements, an improvement in the signal-to-noise ratio can still be achieved. From a clinical application viewpoint, it is crucial to observe at which intervertebral level the nerve conduction is impaired. Current magnetospinography can, at the lowest, achieve a resolution corresponding to the size of one vertebra, that is, an order of 10 mm, which is sufficiently applicable for clinical use, while a resolution of 10-mm order may be insufficient for MEG.

Subsequently, we describe the differences between magnetoneurography and MEG from the perspective of signal source characteristics. Conventional MEG targets signal sources that exist in three-dimensional space within the brain itself. Consequently, the interpretation of reconstructed current distribution obtained from magnetic source analysis can sometimes be a complex task. In contrast, magnetoneurography primarily focuses on signal sources along neural pathways of known anatomical locations, facilitating a relatively straightforward interpretation of the results.

## 7 Limitations

As is the case with the other modalities, the present state of magnetoneurography is characterized by several of limitations. Firstly, the nerve signals that can be applied with this technology are still limited to evoked signals synchronized with stimulation. It cannot target spontaneous neural signals because averaging of thousands of synchronized responses is necessary to sufficiently enhance the signal-to-noise ratio. Furthermore, not all evoked signals can be measured. One issue is that a notable challenge lies in the acquisition of efferent neural signals, attribute to the absence of appropriate stimulation methods to the brain. While transcranial magnetic stimulation (TMS) emerges a potential solution, its application is hindered by the substantial magnetic pulses it generates, which often result in the malfunction of magnetic sensors. Additionally, signals from slow-conducting nerve fibers, like Aδ fibers, have not been successfully detected due to their broad distribution of conduction velocities and rapid dispersion. Further innovation in stimulation methods will likely be necessary.

## 8 Closed-cycle cryocooling system for magnetoneurography

The magnetoneurography based on SQUID magnetometers relies on liquid helium to maintain the superconducting state of the sensors as well as the conventional MEG and MCG. Specifically, the system is designed with a thinner wall for the observation area to bring the SQUID magnetometers closer to the magnetic sources, thereby capturing larger signals. This leads to greater thermal intrusion from the observation area compared to MEG and MCG, resulting in higher consumption of liquid helium. In countries like Japan, where the entire supply of liquid helium is dependent on imports, the cost and supply situation are highly influenced by external factors and unstable. Given the current international circumstance, helium supply often becomes constrained, and its prices are on the rise. The operational costs associated with liquid helium represent a significant financial burden for hospitals and could hinder the widespread adoption of magnetoneurography.

To address the high operational costs due to liquid helium consumption, the magnetoneurograph system has been directly integrated with a helium recondensation system powered by a pulse tube refrigerator (47). In this closed-cycle approach, gaseous helium evaporated from the cryostat is channeled into a recondensation chamber situated adjacent to the magnetically shielded room. There, the pulse tube refrigerator recondenses the helium into its liquid form. The recondensed liquid helium is then transferred back to the cryostat via a high-efficiency transfer tube.

By implementing this closed-cycle helium recondensation system, we have thus far achieved more than 24 months of continuous operation excluding instances such as power outages, with no additional helium refilling required for over a year. While nearly 100% liquid helium recycling has been achieved, the presence of vibration-inducing devices like refrigerator in close proximity raises concerns about their impact on magnetic measurements. However, the mechanical vibrations emanating from the recondensation system adjacent to the magnetically shielded room are below 100 Hz, which does not overlap with the frequency band of signals from the spinal cord and peripheral nerves, allowing for easy removal through filtering. Consequently, measurements can be conducted even while the recondensation system is operational. Additionally, the elimination of weekly refilling downtime not only reduces operational costs but also enhances the convenience of system operation in a clinical setting.

## 9 Conclusions

In this paper, we introduced the methods and applications of magnetoneurography using SQUID magnetometers. As described in Introduction, the first report on magnetoneurography was not made until 1980. This is significantly later compared to eletroneurography, which originated in the 18th century although the discovery of magnetism predates that of electricity by far earlier in human history. The delay can be attributed to the extremely weak magnetic signals obtained from biological sources, as well as the time required for technology to advance to the point where it could detect those magnetic fields. It was only after the establishment of multi-channel biomagnetic measurements using the SQUID magnetometers that the development of magnetoneurography became possible.

In recent years, new magnetic flux sensors, such as optically pumped atomic magnetometers (OPMs), whose magnetic field resolution approaches that of SQUIDs, have been developed, and their application in detecting brain magnetic signals has been reported (19). These new sensors are expected to enable biomagnetic measurements at a lower cost. However, the current OPMs are not sufficient for application to magnetoneurography in terms of their sensitivity, bandwidth, and usability. For magnetoneurography, especially its clinical application, the “old-fashioned” SQUID still holds a clear advantage in both resolution and bandwidth even if they need to be cooled.

As discussed in this paper, magnetoneurography, due to advancements not only in hardware such as magnetometers but also in innovative stimuli and signal processing such as artifact reduction, can now be applied to areas that were previously unmeasurable, paving the way for new clinical applications.

The magnetic field source analysis of magnetoneurography indicated in this paper is entirely based on a spatial filtering method called UGRENS beamformer. It does not take into account the anatomical structure of the body. Additionally, the distribution of volume current is projected onto a two-dimensional curved plane that includes neural pathways. In the future, for magnetic source analysis in cases where the depth of neural pathways changes significantly in three dimensions or when there is a complex conductivity distribution, advanced current source reconstruction methods that consider the anatomical structure of the body will be required. Such new analytical techniques and measurement know-how developed with SQUID-based magnetoneurography can be applied to the next-generation magnetoneurography with new magnetic flux sensors when they progress and achieve at the level suitable for application in the future.
